# Effect of Reactive Ion Etching on the Luminescence of GeV Color Centers in CVD Diamond Nanocrystals

**DOI:** 10.3390/nano11112814

**Published:** 2021-10-23

**Authors:** Sergey A. Grudinkin, Nikolay A. Feoktistov, Kirill V. Bogdanov, Mikhail A. Baranov, Valery G. Golubev, Alexander V. Baranov

**Affiliations:** 1 Center of Information and Optical Technologies, ITMO University, Kronverksky Pr. 49, bldg. A, 197101 St. Petersburg, Russia; grudink@gvg.ioffe.ru (S.A.G.); kirw.bog@gmail.com (K.V.B.); mbaranov@mail.ru (M.A.B.); 2 Ioffe Institute, Polytechnicheskaya 26, 194021 St. Petersburg, Russia; Feokt@gvg.ioffe.ru (N.A.F.); Golubev@gvg.ioffe.ru (V.G.G.)

**Keywords:** diamond nanocrystals, color centers, chemical vapor deposition, photoluminescence, Raman spectroscopy

## Abstract

The negatively charged germanium-vacancy GeV^−^ color centers in diamond nanocrystals are solid-state photon emitters suited for quantum information technologies, bio-sensing, and labeling applications. Due to the small Huang–Rhys factor, the GeV^−^-center zero-phonon line emission is expected to be very intensive and spectrally narrow. However, structural defects and the inhomogeneous distribution of local strains in the nanodiamonds result in the essential broadening of the ZPL. Therefore, clarification and elimination of the reasons for the broadening of the GeV^−^ center ZPL is an important problem. We report on the effect of reactive ion etching in oxygen plasma on the structure and luminescence properties of nanodiamonds grown by hot filament chemical vapor deposition. Emission of GeV^−^ color centers ensembles at about 602 nm in as-grown and etched nanodiamonds is probed using micro-photoluminescence and micro-Raman spectroscopy at room and liquid nitrogen temperature. We show that the etching removes the nanodiamond surface sp^2^-induced defects resulting in a reduction in the broad luminescence background and a narrowing of the diamond Raman band. The zero-phonon luminescence band of the ensemble of the GeV^−^ centers is a superposition of narrow lines originated most likely from the GeV^−^ center sub-ensembles under different uniaxial local strain conditions.

## 1. Introduction

Impurity-vacancy color centers in nanodiamonds (NDs) have attracted the attention of researchers as atom-like emitters for optical quantum technologies, biomedical markers, and temperature nanosensors [1,2,3,4,5,6]. Negatively charged germanium-vacancy centers (GeV^−^) with zero phonon line (ZPL) at a wavelength of ~602 nm at room temperature are among the most promising emitting centers [1,7,8,9,10,11,12,13]. The GeV^−^ center is an interstitial point defect where a germanium atom is positioned midway between two adjacent missing carbon atoms in the diamond lattice with the diamond <111> axis as the principal [1]. This structure is also called a split vacancy configuration leading to a D_3d_ point group symmetry that includes inversion symmetry. A GeV^−^ center has a double orbital degeneracy in both the ground and excited state in this symmetry. The orbital degeneracy is lifted by spin-orbit coupling and dynamic Jahn–Teller interaction, leading to a pair of split ground and excited states [1]. Since GeV^−^ centers have inversion symmetry, they are less vulnerable to electromagnetic fluctuations [14]. The GeV^−^ centers possess superior optical properties: a sharp ZPL (~5 nm at room temperature), a high Debye–Waller factor (with nearly 60% of its emission within ZPL), and a short photoluminescence lifetime of 1.4–5.5 ns [1]. For quantum optics applications, NDs with embedded color centers are desired due to low light scattering and the possibility of their integration with hybrid photonic structure using pick-and-place techniques [15,16,17]. In addition, bright luminescent NDs are suited for bio-sensing and labeling applications as well as for nanothermometry [6].

The GeV^−^ color centers possess the same point group symmetry and similar level structure as the SiV^−^ color centers, thus having comparable optical properties. In general, the emission of GeV^−^ and SiV^−^ color centers strongly depends on crystal strain in the diamond lattice [18,19,20]. In CVD diamond, crystal strains in the diamond lattice are due to edges of crystal boundaries and grain boundaries, dislocations, or other extended and point defects [21]. The color center is susceptible to the local strain field at the center site. Fundamentally, strain affects the electronic energy levels of the GeV^−^ centers by modifying the electronic levels of the GeV^−^ in addition to the spin-orbit and Jahn–Teller interactions [18]. As a result, residual strain in NDs is likely the primary source for the ZPL of color center lineshift [15,20]. Therefore the inhomogeneous broadening of the ZPL of the ensemble GeV^−^ color centers is due to the distribution of the emission wavelengths of individual GeV^−^ centers induced by the lattice local strain in ND.

The heterogeneous spread of the ZPL wavelengths of single emitters makes it challenging to generate indistinguishable photons from different emitters in a single light source, which is necessary to avoid in a few quantum information applications. In particular, the strain gradient in an electrically deflected microcantilever was used to overcome the inhomogeneous distribution of emitter wavelengths and to spectrally align two GeV^−^ centers in bulk diamond film [18]. However, this method is hardly applicable to centers in NDs. At the same time, the use of NDs looks quite attractive since the GeV^−^ centers in nanocrystals demonstrate a ~6 times larger energy split in the ground state than in bulk diamonds due to strain conditions [15]. The large energy split implies a potentially longer spin coherence time in NDs [22]. Therefore, the important problem is optimizing the synthesis and/or post-synthetic processing of NDs with embedded GeV^−^ centers with minimal deformations of the diamond lattice.

In this work, we investigate the spectral properties of ZPL, at about 602 nm, of the ensembles of GeV^−^ color centers in NDs produced by the hot filament chemical vapor deposition (HFCVD) technique and in as-grown HFCVD NDs exposed by reactive ion etching (RIE) in oxygen plasma. We demonstrate, by analysis of as-grown and etched NDs with micro-photoluminescence and micro-Raman spectroscopy techniques at room and liquid nitrogen temperature, that the applied procedure of RIE of NDs allows removal of defective regions in NDs and reduces an inhomogeneous broadening of the ZPL, allowing detailed analysis of the zero-phonon luminescence band of the ensemble of the GeV^−^ centers.

## 2. Materials and Methods

### 2.1. The Fabrication of Nanodiamonds

We realized a top-down method to fabricate NDs with embedded GeV^−^ centers. The starting material was HFCVD grown NDs with in situ incorporated GeV^−^ centers [23]. The parameters of the HFCVD process were as follows: the temperature of the tungsten coil was 2000–2200 °C; the working pressure in the reactor was 40 Torr; the hydrogen flow rate was 500 sccm; the methane concentration was 2%. Before deposition, the substrate was seeded with detonation NDs [24]. Bulk crystalline germanium situated on the substrate holder during CVD growth was used as a solid-state source of Ge atoms. During the HFCVD process, the etching of the solid-state sources of Ge atoms with atomic hydrogen gives rise to the volatile radicals GeH_x_. Germanium atoms being incorporated into the diamond lattice from the gas phase promoted the formation of GeV^−^ center ensembles in NDs. The HFCVD NDs were etched in an oxygen−nitrogen mixture (20/80 vol.%) at the following parameters: microwave power of 250 W (frequency of 2.45 GHz), substrate temperature of 500–600 °C, oxygen−nitrogen mixture flow rate of 100 sccm, and reactor working pressure of 10 Torr. The etching duration of diamond particles was ~15 min.

### 2.2. Microscopy Spectroscopy, Photoluminescence and Raman Spectroscopy

**Figure 1:**
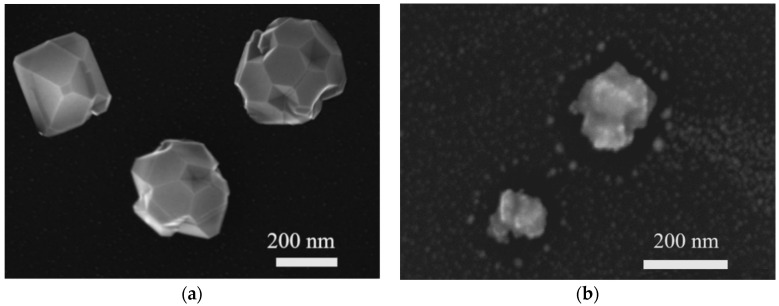
SEM images of as-grown nanodiamonds (**a**) and etched nanodiamonds (**b**). SEM Merlin (Zeiss), accelerating voltage—10 kV with a probe current of 150 pA. The scale bars are shown.

The typical Scanning Electron Microscopy (SEM) images of studied as-grown and etched NDs with GeV^−^ color centers presented in Figure 1a,b, respectively, were obtained with a Zeiss Scanning Electron Microscope, “Merlin” (Zeiss AG, Germany). We secured the samples with carbon tape and created a conductive bridge between silicon, a substrate, and the sample holder. The accelerating voltage was set to 10 kV with a probe current of 150 pA. To achieve better topological contrast, we combined signals from InLens and Everhart-Thornley SE2 detectors (Zeiss AG, Germany). The micro-photoluminescence and micro-Raman spectra were measured in the backscattering geometry using a Renishaw “InVia” Raman spectrometer (Renishaw plc, United Kingdom) equipped with a confocal microscope, Peltier cooled CCD, and 1800 lines/mm grating. The spectral resolution of the spectrometer was ~2 cm^−1^. The excitation laser radiation of 488 nm was focused by a 100× lens (NA = 0.9) into a spot with a diameter of ~2 μm on the selected ND crystal. Thus, both the luminescence and Raman spectra were obtained simultaneously from the same individual ND.

To correctly compare the luminescence intensities of GeV^−^ centers obtained from nanocrystals of different sizes, the luminescence spectra were normalized to the intensity of the diamond Raman line of ~1332 cm^−1^ (521.9 nm), which is proportional to the illuminated nanocrystal volume. Micro-photoluminescence measurements with high resolution were carried out by using 3000 lines/mm grating, which provides a spectral resolution of ~0.8 cm^−1^. Micro-photoluminescence spectra at T = 80 K were measured using a Linkam THMS 600 cryogenic setup. A 50× lens (NA = 0.50) with a large working distance was used in this case to focus the incident beam on the selected individual NDs. All measurements were carried out at least 5 times to prove the reproducibility of the data obtained.

## 3. Results

Figure 1 shows that as-grown HFCVD NDs are sharp, well-faceted crystallites with 250–350 nm grain sizes. The etched NDs have a size of less than 200 nm. The etching effect of RIE is based on the oxidizing reaction and ion bombardment mechanism. The synergetic effect of both treatment mechanisms is that specific areas of the NDs were selectively removed. The change of the NDs’ morphology, compared to starting NDs, is due to the anisotropic nature of RIE in oxygen plasma.

**Figure 2:**
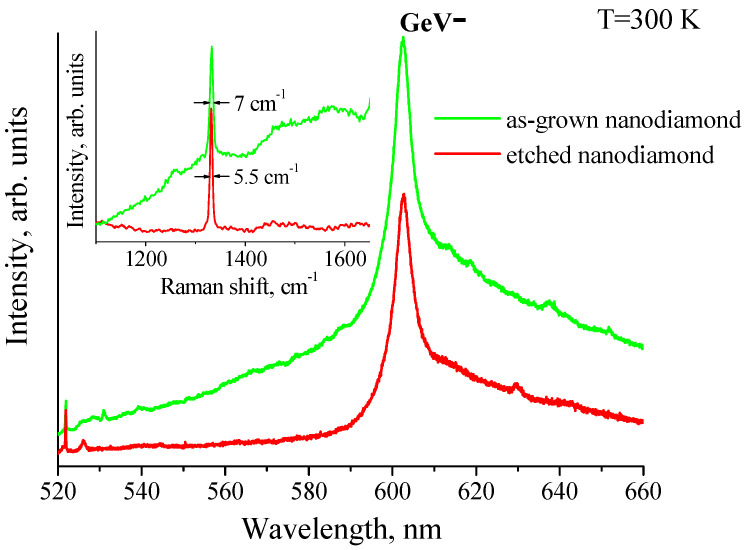
Typical photoluminescence spectra of as-grown nanodiamonds (green) and etched nanodiamonds (red) measured at T = 300 K. The inset shows Raman spectra of the nanodiamonds; the bandwidths of ~1332 cm^−1^ diamond band are shown.

Figure 2 presents the photoluminescence and Raman spectra of as-grown HFCVD NDs and etched NDs. The spectra were measured at room temperature. Raman spectra of the same NDs are seen in the 520–530 nm spectral region. The inset in Figure 2 shows these Raman spectra on an enlarged scale. The photoluminescence spectra contain ZPLs of ensembles of GeV^−^ color centers peaked at a wavelength of ~602 nm with the FWHM of ~6.2 nm. Unlike the photoluminescence spectrum of as-grown NDs, there is a much lower broadband photoluminescence background in the spectra of the etched NDs.

Raman spectra of NDs exhibit the band at ~1332 cm^−1^, corresponding to the TO phonon of F_2g_ symmetry in the diamond lattice (see the inset in Figure 2). Lines with maxima at 1350 cm^−1^ and 1580 cm^−1^ can be assigned to the presence of sp^2−^ hybridized carbon in NDs or/and on their surface [21]. For as-grown NDs, the full width at half-maximum (FWHM) of the Raman line was ~7 cm^−1^, while for etched NDs, the FWHM of the diamond line decreased to ~5.5 cm^−1^. The FWHM was determined with 10% accuracy by the fitting of the Raman band with the Gaussian function. Since strains influence the phonon energies in the diamond lattice, the strain distribution in the NDs leads to a Gaussian broadening of the Raman band. The RIE of as-grown NDs removes the highly defective and, therefore, highly strained surface areas of NDs. The narrowing of the diamond Raman band at ~1332 cm^−1^ indicates the narrowing of the strain distribution in etched NDs. A strong reduction in sp^2−^ hybridized carbon Raman band intensities for etched NDs also supports the supposition that main sp^2^ defect-induced strains are localized in the surface areas of the NDs.

**Figure 3:**
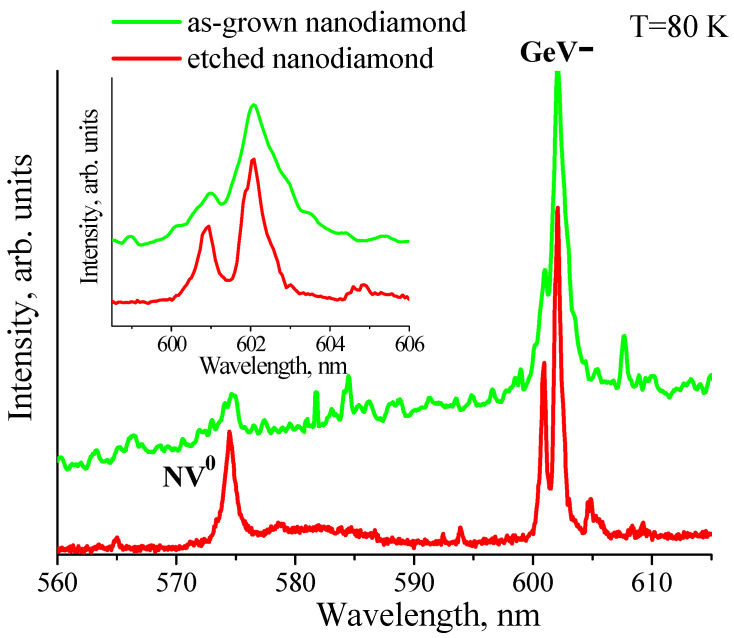
Typical photoluminescence spectra of as-grown nanodiamonds and etched nanodiamonds, measured at T = 80 K. The inset shows in detail a part of the photoluminescence spectra in the vicinity of the ZPL of GeV^−^ color centers. The attribution of the luminescence lines to different color centers is indicated (GeV^−^ and NV^0^).

The normalized photoluminescence spectra of as-grown HFCVD NDs and etched NDs at T = 80 K in the spectral range of 560–620 nm are shown in Figure 3. The ZPLs of GeV^−^ center ensembles split into doublets due to the spin-orbit splitting of the excited state of the GeV^−^ center [12]. The phonon sideband accompanying the ZPL has very low intensity. The linewidths of the doublet in spectra of as-grown NDs are broader than the linewidths in spectra of etched NDs (see the inset in Figure 3). The narrowing of the ZPL agrees with the Raman data above and suggests that the RIE is responsible for removing the most defective strained surface regions with GeV^−^ centers.

An additional, albeit indirect, argument confirming this conclusion is the significantly higher intensity of the ~575 nm band observed at 80 K in the photoluminescence spectra of etched NDs as compared to as-grown ones (Figure 3). This line, corresponding to a neutral nitrogen-vacancy color center (NV^0^) in diamond [25], was not observed at room temperature. This result is due to strong quenching induced temperature-dependent non-radiative recombination processes controlled by defects in proximity to NV^0^ color centers [26]. This implies that the NV^0^ center line’s greater intensity for etched NDs is associated with the removal of the defects during RIE. It should be noted that the signal of NV^−^ centers at 637 nm was not clearly observed in spectra, most likely due to photoconversion of NV^−^ centers to NV^0^ centers observed in CVD NDs under excitation at a wavelength of less than 490 nm [26,27].

**Figure 4:**
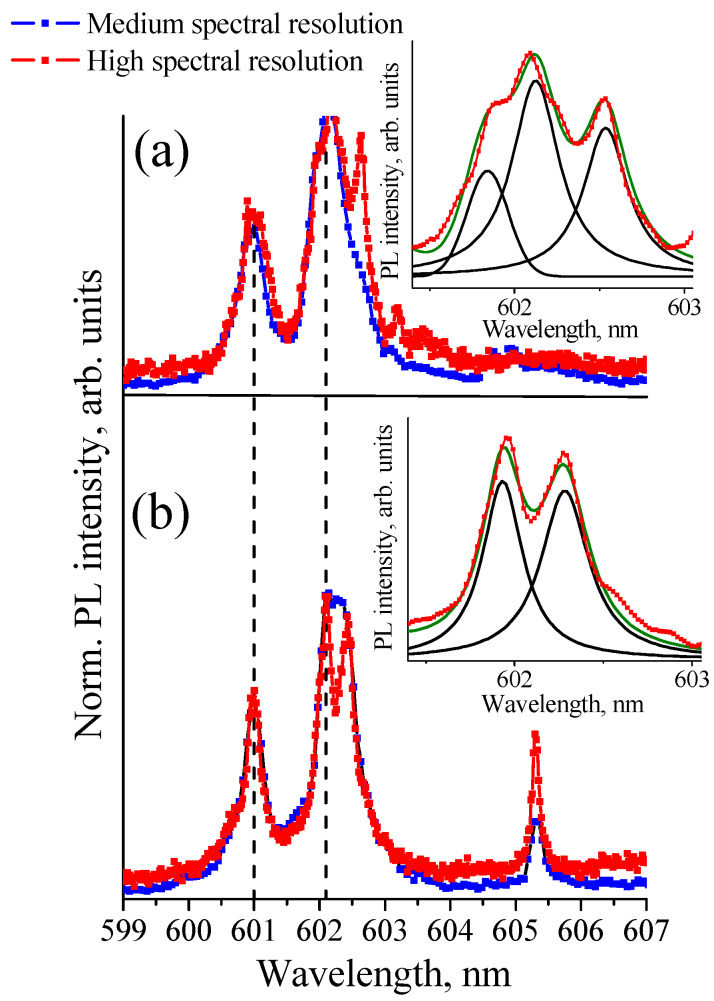
The ZPL spectra of the ensembles of GeV^−^ color centers. The spectra of two different nanodiamonds shown in (**a**,**b**) were recorded with medium (0.07 nm) and high spectral resolution (0.03 nm) at T = 80 K. Inset: Deconvolution of the broad line of the ZPL doublet with the maximum at ~602.1 nm. The vertical lines show the spectral positions of the ZPL doublet of GeV^−^ centers for microcrystal diamond with high crystalline quality at T = 77 K [12].

Even in etched NDs with a reduced number of defects, the spectra of ZPLs of the ensembles of GeV^−^ color centers seem to be inhomogeneously broadened by the distribution of the local strain fields. The fine structure of ZPL spectra is clearly seen in ND micro-photoluminescence measurements with high spectral resolution. Figure 4a,b shows ZPL spectra of two different NDs measured with a spectral resolution of 0.07 nm (2 cm^−1^, medium resolution, blue line) and 0.03 nm (0.8 cm^−1^, high resolution, red line).

**Figure 5:**
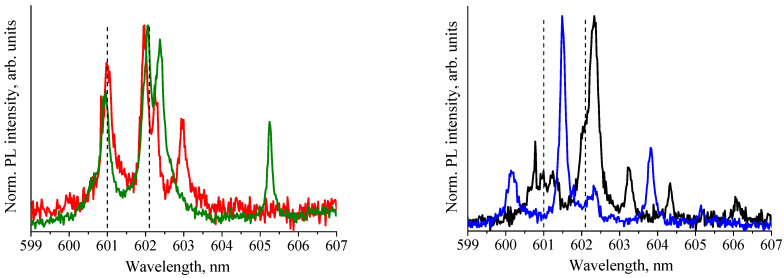
High-resolution ZPL spectra of the ensembles of GeV^−^ color centers for various etched nanodiamonds demonstrating markedly different spectral positions and intensities of the ZPL doublets. The spectra were recorded at T = 80 K. The vertical lines show the spectral positions of the ZPL doublet of GeV^−^ centers for microcrystal diamond with high crystalline quality at T = 77 K [12].

For comparison in Figure 4, vertical lines show the spectral positions of the ZPL doublet of GeV^−^ centers found earlier for microcrystal diamond with high crystalline quality at T = 77 K [12]. High spectral resolution measurements made it possible to establish that the broad line of the ZPL doublet with the maximum at ~602.1 nm consists of either three narrow lines at ~602.0, ~602.2, and ~602.6 nm for the spectrum shown in Figure 4a, or two lines at ~602.0 and ~602.3 nm for the spectrum shown in Figure 4b. The insets in Figure 4 show deconvolution of the broad line of the ZPL doublet with the maximum at ~602.1 nm in Gaussian lines. Thus, the doublet structure of the zero-phonon-line spectra of the ensemble of the GeV^−^ centers is a superposition of several narrow lines. We attribute these lines to single GeV^−^ color centers and/or GeV^−^ centers’ sub–ensembles. We speculate that, by analogy with the SiV^−^ in nanodiamonds [27], the spread of the local strain within nanodiamond is such that an ensemble of color centers can be represented as a set of sub-ensembles of GeV^−^ centers located in regions with different local strain fields. At the same time, the GeV^−^ centers belonging to one sub-ensemble experience practically the same local strain field. Then, the largest ZPL shifts relative to the high crystalline quality microcrystalline diamond correspond to the most strained areas of NDs. Different local strain values lead to the appearance of a set of ZPL lines with different wavelengths in the ND luminescence spectra, as shown in Figure 4 and Figure 5, where the spectra of various etched NDs were obtained at T = 80 K with a high spectral resolution are shown.

For various NDs, the spectral positions of the ZPL doublets are dispersed in the range of 600–604 nm (Figure 4 and Figure 5). The narrow luminescence lines are also observed in the spectral range of 603–609 nm. We attribute these usually weak lines to small sub-ensembles of GeV^−^ centers localized in highly strained regions. A similar observation for the ensemble of the SiV^−^ centers was reported by Neu et al. demonstrating that an inhomogeneous spectral broadening ZPL of an ensemble is accompanied by an asymmetric tail toward longer wavelengths [27].

## 4. Discussion

Strain aligned along the [111] direction can shift the energies of the ground and excited states of the GeV^−^ center, and this can appear as a shift in the position of the ZPL. Strain transverse to the [111] direction does lift the orbital degeneracy, leading to an increase in the splitting between fine-structure lines in the ZPL [16]. In ZPL spectra, the lines of transitions between split ground and excited states are homogeneously broadened due to the electron−phonon coupling. The excited state splitting value (4.5 meV) is higher than the ground state splitting value (0.7 meV) [1]. Therefore, at T = 80 K, the ZPL width is narrow enough to resolve only the doublet, due to excited state splitting, but the doublet, due to ground state splitting, is not observed. Overlapping ZPLs of different sub-ensembles of GeV^−^ centers did not make it possible to determine a change in the value of the excited state splitting caused by the local strain field in NDs.

The main mechanisms that lead to the color center wavelength shift are hydrostatic pressure and uniaxial strain. It was found by Lindner et al. that for SiV^−^ centers in the NDs, hydrostatic-type pressure results in a blue shift of the ZPL. In contrast, uniaxial strain causes a larger redshift with different magnitudes depending on the direction of the strain [20]. The same strain influence is expected for GeV^−^ centers, having the same structure as SiV^−^ centers. Comparative analysis of the ZPL spectra in Figure 4 and Figure 5 shows that a redshift of the ZPLs of sub-ensembles of the GeV^−^ centers is significantly more likely than a blue-shift. We believe that analogously to the SiV^−^ case in HFCVD NDs, the distribution of uniaxial strains induces the shifts of ZPLs of sub-ensembles of GeV^−^ centers. A blue-shift of the ZPL in the spectrum in Figure 5 (blue curve) may be because it is the spectrum of an aggregate of NDs, i.e., several intergrown NDs. The competitive growth of NDs can cause the appearance of hydrostatic pressure in an aggregate of NDs.

## 5. Conclusions

We realized a top-down method to fabricate NDs with embedded GeV^−^ centers. The NDs with GeV^−^ color centers were produced by the hot filament chemical vapor deposition (HFCVD) technique on silicon followed by reactive ion etching in oxygen plasma. We showed that the reactive ion etching in oxygen reduces the number of structural defects localized in the surface area of the NDs; this resulted in the Raman line of diamond narrowing to 5.5 cm^−1^ and increased the luminescence intensity, corresponding to neutral nitrogen-vacancy color center NV^0^. The cooling of the samples to the liquid nitrogen temperature enables us to observe the fine structure of the inhomogeneously broadened ZPL of ensembles of the GeV^−^ centers in the strained nanodiamonds, using the high-resolution micro-photoluminescence technique. The origin of inhomogeneous broadening of the ZPL is speculated to be due to the fact that the observed ZPL spectra of the GeV^−^ are overlapped with ZPLs of color center sub-ensembles under different strain conditions. At the same time, the color centers belonging to the one sub-ensemble experience practically the same local strain field. The strain response of ZPL spectra of the ensembles of GeV^−^ at T = 80 K and its analysis provides a way to image the strain distribution in CVD nanodiamonds optically.

## Data Availability

The data presented in this study are available within this article. Further inquiries may be directed to the authors.
